# What Are Fungal Infections?

**DOI:** 10.4084/MJHID.2011.001

**Published:** 2011-01-14

**Authors:** Ben E. de Pauw

**Affiliations:** University Medical Center St Radboud. The Netherlands

## Abstract

Yeasts and moulds now rank amongst the 10 most frequently isolated pathogens in febrile patients with an impaired immune system. Fungi are mainly opportunistic pathogens that only invade the body if a severely weakened natural defense permits them to do so. Most factors facilitating an invasive fungal infection are unavoidable because they are directly connected to the underlying diseases as well as to their treatment.

Modern aggressive treatment modalities jeopardize the defense mechanisms to an extent that even fungi with a low virulence may enter the body.

## Introduction

Most of the time, the human species live in peaceful coexistence with the microorganisms that surround them and only when the defense system is damaged or the concentration of pathogens reach an exceptionally high density, an infection may emerge. Most infections pass by unrecognized but sometimes the infecting agents do elicit a response of the body, which leads to clinically manifest signs and symptoms, a condition known as infectious disease. Bacteria, viruses, parasites, fungi, prions, worms, helminthes have all been incriminated in infectious diseases, of which those caused by common viruses are the most frequent, and, until a few decades ago, those by bacteria the most feared. As strategies to control bacterial infections in patients improved, fungi became the most hazardous pathogens. Yeasts and moulds now rank amongst the 10 most frequently isolated pathogens among patients in Intensive Care Units. Approximately 7 percent of all febrile episodes that occur during neutropenia can be attributed definitely to invasive fungal infections. *Candida* has become the fourth leading bloodstream isolate in hospitals in the USA, surpassing many historically notorious bacterial pathogens. Since the eighties an increased incidence of invasive fungal infections in patients who are not in an end stage of their underlying disease was observed.^1^ Moreover, due to the ubiquitous low autopsy rate their incidence is probably underestimated because signs and symptoms are seldom characteristic, which makes that many invasive fungal infections are not detected while the patient is alive.^2^

Why fungi did evolve to become such formidable enemies of seriously ill patients? First of all, it is clear that fungi, in spite of their potential to make bread rise, to provide us with beer and wine, and to give taste to cheese and other food, primary belong to the end of biological life. They are active in disposing decaying organic material, including human corpses, which will eventually be dissolved by fungi, the big cleansing machines of the world. Signals of decay appear to trigger fungal growth and, once activated, the fungus will not stop expanding just because medical interventions aim to postpone a physiological death. On the contrary, modern treatment modalities may even facilitate the growth of fungi through negative interference with the remaining components of the immune system. Let’s have a closer look at these peculiar infective agents, called fungi or mycoses.

## Mycology

**Figure 1:**
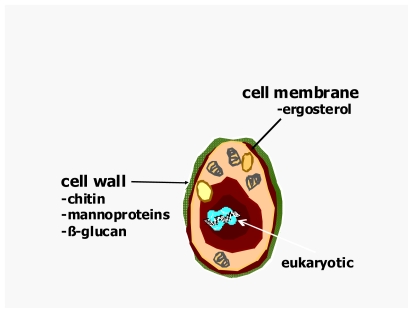
The Fungus

Fungi are remarkable organisms and they constitute a separate kingdom for purposes of classification. Fungi are eukaryotes; they have a membrane surrounding their nucleus, their cells are much larger than bacteria and their molecular processes closely resemble those of plants and animals. However, unlike mammalian cells, fungi almost always possess a rigid cell wall composed of chitin products that surrounds their plasma membrane (see figure 1). A fungus is a vegetative organism and is definitely not a plant either because fungi do not synthesize chlorophyll. It is non-motile life form and its basic structural unit consists of either a chain of cylindrical cells (hyphae) or an unicellular form, or both. The most common species like *Aspergillus* and *Candida* are found everywhere on earth. Gardens, playgrounds, houses, hotels, hospitals and even the skin and mucous membranes have been identified as sources of fungi that caused life-threatening infections.

In the course of time more than 69.000 species of fungi have been described while it has been estimated that the total number of fungal species is of the order of 1.5 million. They range from giant puff-balls mushrooms - sometimes edible, often poisonous - to moulds and yeasts only visible by microscope. Moulds are known to have caused massive economic losses and even food shortage when they infected and destroyed food crops disguised as mildew and rust. Since only a limited number of fungi are regular causes of human pathology, the number of seemingly obscure names and terms that appear in the literature appear to be unnecessarily huge. Furthermore, more than one word is every so often used to describe one and the same thing. Partly as a result of the purportedly complex mycological terminology, clinicians feel seldom tempted to extend their field of interest to detailed study of fungal pathogens, leaving mycology to laboratory scientists. However, if a clinician would only take a few minutes, he would be paid back with an insight in the mechanisms that make fungi what they are, a danger to very vulnerable patients after a sophisticated medical intervention. Indeed, very few mycological terms need to be captured in order to understand the principles of diagnosis and management of mycoses. Just a handful of definitions clarifies the ratio behind the human observations and deliberations that form the basis of fungal classification.

Fungi, like all living things, are recognized and identified on the basis of their shapes, structures and their behavioral properties. Fungi that exist predominantly in the form of independent single cells are usually called *yeasts* while those based on hyphal threads are called *moulds* (i.e. hyphal fungi). Hyphae and yeast are nearly always microscopic cell forms. A complex of hyphal strands, hyphal branches and any associated spore-bearing structure is known as a *mycelium*. Vegetative growth of both yeast and hyphae occurs by means of mitotic, asexual cell division, typically outgrowth of a daughter-burst from an existing fungal cell. Most fungi are also capable of meiotic, sexual reproduction. Matings may occur not only between two different fungal strains, but even between different cell units within a hypha or between mother and daughter yeast cells; instances of fungal parthenogenesis are also known. The result of meiosis in fungi is the formation of a sexual spore. Fungal species and strains that are incapable of sexual reproduction are called *deuteromycetes* o *hyphomycetes*, previously defined as *fungi imperfecti*. A cell unit within a fungal hypha is segregated, although not fully, from its neighbor by a cross-wall or septum. The hyphae of zygomycetes have no or only very few septa. Some species amongst the zygomycetes shed large collections of asexual spores inside a rounded sac, a sporangium, and these spores are known as sporangiospores. A spore that is not surrounded by a sporangium is nowadays referred to as a conidium. Conidia are produced on or in a conidiophore that can range from an undifferentiated hyphal cell to a tissue complex visible to the naked eye. Most yeasts vegetate by a budding process, thereby forming a blastospore. For practical reasons practioners like to lump mycoses of similar origin under single headings. One of the broadest collective mycosis names so far invented is phaeohyphomycosis, which refers to any infection caused by a darkly pigmented (dematiaceous) mould. Its antonym, hyalohyphomycosis, has become popular to describe any infection caused by a colourless mould. Dermatomycosis refers to any type of fungal infection involving the skin, while the term disseminated mycosis describes a fungal infection that spreads to involve at least two deep organs and/or the skin. Some fungal pathogens need to change their cell shape to allow invasion: such *dimorphic* pathogens usually switch from a mould form in their natural environment to a budding, round-celled form in tissue. *Blastomyces dermatitidis, Histoplasma capsulatum, Paracoccidioides brasiliensis*, and *Sporotrix schenckii* are the best-known representatives of the dimorphic manifestation. Other less common fungal pathogens behave similarly and many fungi display subtle morphological differences between forms found in tissues and shapes encountered in cultures. *Coccidioides immitis* converts from a mould form in the environment to a unicellular spherule containing sporangia in infected tissue. 3

**Figure 2:**
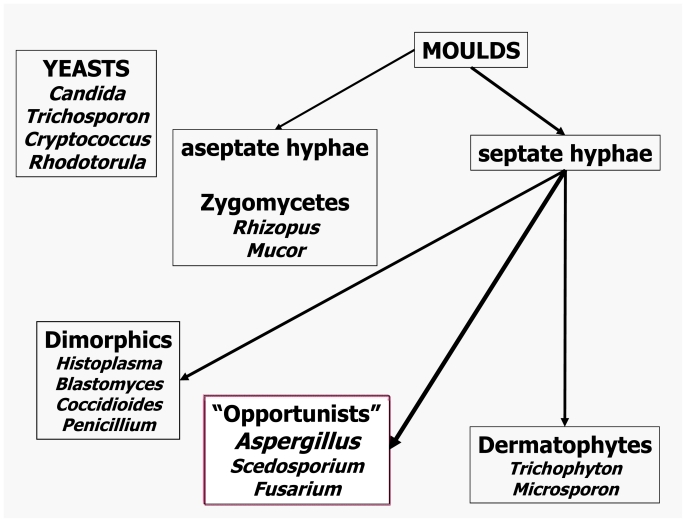
Classification of Fungi

A simplified, schematic classification of the most relevant pathogenic fungi is provided in figure 2.

## Pathophysiology of fungal infections

Only a few of the fungi pathogenic for humans are sufficiently virulent to infect a healthy host. Most are relativel harmless unless they encounter an immunocompromised patient, in whom a weakened defence system permits them to invade the body. Under normal circumstances, the intact epithelial surfaces of the gastrointestinal tract will prohibit invasion by micro-organisms and the mucociliary barrier of the respiratory tract prevents aspiration of fungal cells and spores, while, in contrast, dead or damaged tissue may turn into a breeding ground for infection. For these reasons invasive fungal infections have to be ranked amongst the typically opportunistic infections.

Recently it has been hypothesized that the susceptibility to invasive fungal infections is influenced by genetic variation within key innate or adaptive immune response genes that may lead to a failure in the IL-10 production, Toll-like receptor polymorphism, plasminogen gene polymorphism, etc.^4^,^5^ These data are very intriguing but for the time being severe damage to one or more components of the immune systems appears to be the most important risk factor. As a consequence of treatment-related injury of all natural defense systems, invasive fungal infections will occur in patients who are treated for a hematological malignancy. See table 1. High doses of chemotherapeutic agents not only cause neutropenia, they also inflict serious damage to the mucosal barrier, resulting in impaired production of saliva, secretory IgA, mucus and gastric acid, malabsorption, as well as decreased peristalsis.^6^ Debilitating mucosal damage and the widespread introduction of central venous catheters disrupt the integument of patients. Of course, neutropenia remains a crucial factor that predisposes for fungal infections but prolonged use of even moderate doses of corticosteroids is equally detrimental through both impairment of the T-cell function and alteration of the glucose metabolism.^7^,^8^ In case of coincidence of these factors any potential pathogen that has invaded damaged tissue may gain easy access to the body and, subsequently, disseminate rapidly. A recent study highlights that even patients with hematological malignancies, undergoing a nonmyeloablative allogeneic “minitransplants” to reduce transplant-related toxicity, are at high risk of contracting an invasive fungal infection.^9^ Cytotoxic T-cells as well as the immunosuppressive agents used to control graft-versus-host disease may damage both the mucosal barriers and the cellular immunity. Therefore, the high incidence of invasive fungal infections in bone marrow transplant recipients relative to those who received conventional cytoreductive chemotherapy is not a surprising finding. Over the last decennium the number of bone marrow transplantation procedures has tripled in the western world, but the position of allogeneic transplantation in the cutting edge of prime bringers of invasive fungal infections in critically ill patients is at risk. With the growing use of powerful immunosuppressive purine analogues, fludarabine, and cladibrine, as well as employment of anti-T and anti-B cell antibodies in the conventional management of lymphoreticular and other hematological malignancies, the number of patients at risk will rise to similar levels.^10^ In addition, improved supportive care has fostered the application of more intensive chemotherapeutic regimens for malignant diseases to elderly individuals and patients with pre-existing immunocompromising diseases such as diabetes mellitus and chronic obstructive pulmonary disease. Where the hematological malignancies have served as a role model in the recognition of invasive fungal infections, other diseases, both malignant and non-malignant, that are associated with intrinsic or treatment-related immunodeficiencies followed suit.^11^ In these populations the incidence of invasive opportunistic nosocomial infections has more than doubled over the last decades, because of advances in cytotoxic therapy, improved treatment of bacterial infections, the arrival of AIDS, and, most of all, the introduction of modern, very potent immunosuppressive agents.^10^,^11^ In addition to the significant contribution of fungal infections to the morbidity and mortality in immunosuppressed patients, an estimated 15 percent of the world’s population is infected with superficial dermatophyte fungi. There is even an increase in the frequency of this type of fungal infections due to life-style changes such as more intense use of swimming pools and health spas and crowding conditions in nursing homes, schools and prisons.

Virtually all human fungal infections originate from the environment through skin contact with or without trauma, or via inhalation or ingestion of fungal spores. *Candida,* the prototype of a colonizing fungus that may stay unnoticed on the surfaces of a body has become the most common genus of fungal pathogens and remains very menacing. Nowadays candidemia has become a well recognized threat in hematological patients with chemotherapy-induced granulocytopenia, notably in those with concurrent severe mucosal damage and when venous access devices are in use. *Candida* species are a normal part of the microflora of the oropharynx and the gut which explains why alterations in the equilibrium between the indigenous organisms by the use of broad-spectrum antibacterials can lead to overgrowth of *Candida* species. The fact that the gut is a major source of disseminated candidosis is increasingly acknowledged.^6^, ^12^ Gastrointestinal colonization is recognized as the most important risk factor for candidemia, perhaps with exception of infections by *Candida parapsilosis*, an organism that is commonly found on the skin and has been identified as one of the leading causes of venous catheter-related fungal infections. Candidemia tends to develop rather early during the treatment course and only 10 percent of the infections emerge outside the hospital. The incidence of catheter-associated fungemia varies from 1–16 percent and depends on the presence of the recognized risk factors. Whilst the incidence of *Candida* infections declined since the late 1970s, an increase in the rate of pulmonary aspergillosis was observed. 2,13 Some even reported an incidence as high as 40 percent amongst recipients of sibling marrow. *Aspergillus* species, notably *Aspergillus fumigatus*, are undoubtedly prominent and deadly fungal pathogens. *Aspergillus* species are airborne pathogens that pass through the nose, may penetrate into the paranasal sinuses and will eventually land in the lower respiratory system that constitutes the major port-d’entrée. The inevitable risk factors, such as graft-versus-host disease, high dose corticosteroid use, and recurrent prolonged neutropenia explain the increasing rate of pulmonary aspergillosis as well as the bimodal time distribution curve of the development of invasive aspergillosis amongst recipients of allogeneic stem cell transplants.^14^,^15^ Approximately 40 percent of the cases of aspergillosis will surface within a few weeks after transplantation, i.e. before engraftment. The second peak of incidence occurs after 100 days or more when typically graft-versus-host disease necessitates the employment of high doses of corticosteroids and ganciclovir treatment of cytomegalovirus reactivation reintroduces neutropenia.^15^ After marrow engraftment and mucosa healing, the incidence of fungal infections diminishes accordingly until the cell-mediated immunity has fully recovered. This may require quite some time, especially in case of chronic graft-versus-host disease.

During the last decades an obvious change in pathogens has occurred. More aggressive treatment modalities can jeopardize the defense mechanisms to an extent that even fungi with a low intrinsic virulence enter the body to cause serious disease. Organisms that were previously considered harmless commensals have been held accountable for serious invasive fungal disease. Non-*Aspergillus* moulds like Zygomycetes can generate a clinical picture indistinguishable from *Aspergillus*.^16^ *Fusarium*, a soil fungus, can enter the body through the respiratory system or via severe onychomycosis but has also been connected with venous access devices, its prevalence being clearly higher in patients who carry a central venous line.^17^ *Scedosporium*, another emerging pathogen, is being increasingly isolated from patients with a persistently impaired cellular immunity in combination with a protracted granulocytopenia.^18^,^19^ In 1984, 80 percent of *candida* bloodstream infections were due to *Candida albicans*, whereas in the 1990’s non-albicans species have become responsible for at least half of these infections in patients treated for hematological malignancies.^20^ Both the decrease in the number of *Candida* infections and the shift towards non-*Candida albicans* strains is presumably linked with the use of fluconazole for prophylaxis. Obviously, suppression of the predominant and virulent *Candida albicans* subpopulation created space for less susceptible organisms.

## Diseases caused by invasive fungal infections

A comprehensive list of all fungi that have been incriminated as opportunistic human pathogens may well exceed 400 species, even if the list is restricted solely to those species for which evidence of infection is available. Many of the species listed would have been encountered clinically on very few occasions indeed –often only once or twice- so that fewer than 100 fungal species approach the status of regular human pathogens. The characteristics of all invasive mycoses that have been reported in the literature during the last 25 years are summarized in table 2. Invasive fungal infections can be divided in two distinct groups: The endemic or dimorphic mycoses that are caused by true pathogenic fungi as compared with the opportunistic mould and yeast infections that are saprophytes, which only will invade an immunocompromised host. Histoplasmosis, coccidioidomycosis, blastomycosis and paracoccidioidomycosis are referred to as the endemic mycoses because they used to be geographically confined to certain regions on the American continent. Similarly, *Penicillium marneffei* was typically seen in Southeast Asia. However, as a result of increased travel activities these infections can now occur anywhere. Candidiasis and aspergillosis are the most common diseases caused by opportunistic fungi. 21,22

Invasive *Candida* infections originate most frequently from endogenous reservoirs in patients with lowered host defense but exogenous infections in hospitalized patients are frequently transmitted via the hands of health care workers. Deep-seated invasive infections tend to occur principally in two different patient populations: granulocytopenic patients with a hematologic malignancy and, alternatively, intensive care patients after multiple abdominal surgery, with central venous lines, renal failure and treatment with broad spectrum antibiotics.^7^ Discrimination between colonization and invasive *Candida* infection can be very vexing in the febrile unstable intensive care patient who shows negative blood cultures but has *Candida* cultured from non-sterile body sites. Secondary bloodstream infections, which evolve from other infectious foci such as wounds, carry a much higher mortality than do primary, catheter-related infections.^23^, ^24^. Some patients present with sudden onset of fever, tachycardia, tachypnea, occasionally accompanied by chills and hypotension during treatment with broad spectrum antibacterials. Additional clinical clues that should lead to a suspicion of *Candida* infection include unexplained polymyalgia, polyathralgia, and azotemia. In about 10 percent of patients with acute disseminated candidiasis characteristic pinkish-purple, not tender subcutaneous nodules may arise anywhere on the body. Biopsy specimens should be cultured and, additionally, meticulously screened histologically at multiple levels in an attempt to establish a final diagnosis. Clinical symptoms of *Candida* ophthalmitis seldom occur in neutropenic patients since the lesions and complaints are the result of an inflammatory response of granulocytes. Other patients show an insidious onset of their candidemia, in some cases due to concurrent corticosteroids that suppress fever; they feel initially relatively well until the infection progresses and organ failure becomes evident. Disseminated candidiasis may mimic a single organ infection but in most cases a number of organs are affected. Primary *Candida* pneumonia is regarded as exceptional, whereas the frequency of pulmonary involvement in disseminated infection may be as high 50 percent. The attributable mortality from candidemia ranges from about 40 percent in patients with candidemia alone to over 90 percent for those who have acute tissue invasion with or without fungemia. Many patients die within the first 48 hours after establishing the diagnosis.^25^ There does not seem to exist a substantial difference in outcome between severely ill neutropenic and non-neutropenic patients. Within the category of patients who had been treated for a malignancy, however, a return of the granulocytes is an important prognostic feature.

The most common initial presentation of invasive aspergillosis is unremitting fever accompanied by development of lung infiltrates despite broad-spectrum antibacterial treatment, typically after the second week of chemotherapy-induced neutropenia or highly dosed corticosteroids.^26^ In approximately 90 percent of cases the lungs are the portal of entry, with nasal sinuses and the skin accounting for the remainder of cases. Clinicians should suspect the diagnosis in a patient with pleuritic pain, hemoptysis, localized wheezing and rubbing, or radiographic evidence of a pleural effusion or localized pulmonary infiltrates. The symptoms are due to fungal invasion causing extensive necrosis and occlusion of small blood vessels and resemble those of a pulmonary infarction. A distinct halo of low attenuation surrounding the lesions and a so-called air crescent sign on a chest radiograph or computerized tomography are generally regarded as characteristic of aspergillosis.^26^ The majority of cases of invasive aspergillosis are treated on a presumptive diagnosis because invasive diagnostic procedures are either unavailable, unreliable or precluded by concomitant thrombocytopenia. For practical purposes it is generally accepted that isolation of *Aspergillus* species from clinical specimens such as bronchial washings in the neutropenic patient connotes either invasive infection or a high risk of developing invasive disease shortly. It has to be underscored that it is important that even after start of systemic antifungal therapy, a definite diagnosis remains to be pursued and that infections by other pathogens such as *Actinomyces*, Mycobacteria, *Nocardia*, and other rare fungi are excluded as much as possible. A questionable diagnosis will keep nurturing uncertainty among attending physicians about the optimal therapeutic strategy, particularly if the patient is not responding optimally or if serious adverse events arise. A final diagnosis will also have substantial prognostic value as well as implications for long-term treatment. Whereas the response rate of patients with persisting unexplained fever to systemically active antifungals is about 80 percent if neutropenia resolves, the successful outcome of documented invasive fungal infection may, even under optimal circumstances, not exceed 20 percent.^27^,^28^ This figure is greatly influenced by the state of the underlying disease and possible recovery of the granulocytes and cellular immunity.^29^

## Figures and Tables

**Table 1:** Factors promoting invasive fungal infections

Granulocytopenia
Depressed cellular immunity -viral infections, e.g. cytomegalovirus - a.o. corticosteroids, cyclosporin, antithymocyteglobulin, total nodal and total body irradiation -cytotoxic drugs (cyclophosphamide), purine antagonists
Mucosal barrier injury
Poor hygiene
Genetic predisposition
Use of antibacterials, manipulation of a patient’s microbiological flora
Increasing age of patients with co-morbidity
Use of H2 receptor antagonists
Central venous lines with or without hyperalimentation
Recent gastrointestinal surgery
Neonatal intensive care – low gestational age, low Apgar score, length of stay, intubation

**Table 2:** Invasive fungal infections; pathogens and characteristics of disease.

OPPORTUNISTIC PATHOGENS		
Disease type	Causative agent	Clinical signs and symptoms

Candidiasis	*Candida* spp	Acute disseminated: fever, chills, polymyalgia, polyarthralgia, not tender pinkish skin lesions, retinal exudates. Chronic: complaints of the organ involved.

Aspergillosis	*Aspergillus* spp	Unremitting fever and pulmonary infiltrates during antibiotic therapy. Chest pain, pleural rub, pleural effusion, hemoptysis. Halo and air crescent sign on chest radiograph and CT scan. Clinical and radiologic sinusitis.

Cryptococcosis	*Cryptococcus neoformans*	Flu-like symptoms; skin lesions, headache without meningismus.

Zygomycosis	*Rhizopus* spp *Absidia* spp *Mucor* spp	Like aspergillosis, more outspoken rhino-cerebral form with serosanguinous nasal discharge.

Others	*Malassezia furfur*	Often catheter-associated; pneumonia
	*Trichosporon* spp	Skin and lung lesions
	*Fusarium* spp	
	*Pseudallescheria boydii*	Often positive bloodcultures. Skin lesions, severe myalgia. Abscess formation with symptoms depending on organ involved.
	*Scedosporium* spp	Like aspergillosis; wound infections.
	*Alternaria* spp	

**ENDEMIC PATHOGENS**		

**Disease type**	**Causative agent**	**Clinical signs and symptoms**

Blastomyscosis	*Blastomyces dermatitidis*	Ulcerative lesions; skin, urogenital tract
		Central nervous system

Histoplasmosis	*Histoplasma capsulatum*	Pulmonary infiltrates; mucocutaneous ulcers
		Hepatosplenomegaly

Coccidioidomycosis	*Coccidioidis immitis*	Pulmonary infection. Dissemination with osteomyelitis, arthritis, meningitis.

Para-coccidioidomycosis	*Paracoccidioidis brasiliensis*	Pulmonary infection. Dissemination to skin, mucosa and lymphnodes.

Penicilliosis	*Penicillium marneffei*	Skin and subcutaneous laesions, lung, lymphadenitis, splenomegaly.
